# CALL FOR ACTION: STRENGTHENING LONG-TERM CARE IN NEPAL

**DOI:** 10.1093/geroni/igac059.2860

**Published:** 2022-12-20

**Authors:** Usha Dhakal, Sara McLaughlin

**Affiliations:** Miami University, Oxford, Ohio, United States; Miami University, Oxford, Ohio, United States

## Abstract

This paper aims to introduce the existing long-term care (LTC) system in Nepal, identify key emerging issues, and provide possible policy recommendations for addressing weaknesses in the current system. Although the majority of older Nepali adults rely on their adult children for care, this traditional care arrangement is weakening for a variety of reasons. As a result, older Nepali adults increasingly seek care elsewhere such as “old age homes” (OAH), which generally meet only their basic needs. More recently, adult day centers have been established that enable older Nepali to receive help with personal care and instrumental tasks and/or health care needs in the absence of their families; however, they are primarily located in big cities and largely limited to those who can afford to pay. With a growing older population and societal changes that make familial care increasingly challenging for adult children, the Government of Nepal must foster the development of a sustainable system of LTC. This will necessarily involve building and sustaining a skilled geriatric workforce, capitalizing on the natural “villages” that have existed in Nepal for hundreds of years, and standardizing and monitoring the operation of LTC facilities. Additionally, public service campaigns to help destigmatize the use of OAH and day centers and integration of home and community-based services to make care arrangements locally could help make these newer LTC approaches more acceptable. Finally, nationally representative studies aimed at understanding the health and care needs of Nepal’s rapidly increasing older population is of utmost priority.

